# Evolving Paradigms in the Management of Trigeminal Nerve Injuries Post Oral Surgery: A Comprehensive Narrative Review

**DOI:** 10.7759/cureus.88631

**Published:** 2025-07-23

**Authors:** Saanvi Tank, Amit Patil, Tejal Patil, Minal M Kshirsagar, Aarti S Bedia, Sanpreet S Sachdev, Vyshnavi Mundada

**Affiliations:** 1 Conservative Dentistry and Endodontics, Bharati Vidyapeeth (Deemed to be University) Dental College and Hospital, Navi Mumbai, Mumbai, IND; 2 Oral And Maxillofacial Surgery, Bharati Vidyapeeth (Deemed to be University) Dental College and Hospital, Navi Mumbai, Mumbai, IND; 3 Public Health Dentistry, Bharati Vidyapeeth (Deemed to be University) Dental College and Hospital, Navi Mumbai, Mumbai, IND; 4 Oral Medicine and Radiology, Bharati Vidyapeeth (Deemed to be University) Dental College and Hospital, Navi Mumbai, Mumbai, IND; 5 Oral Pathology and Microbiology, Bharati Vidyapeeth (Deemed to be University) Dental College and Hospital, Navi Mumbai, Mumbai, IND

**Keywords:** microsurgical repair, nerve regeneration, neurosensory testing, oral surgery, trigeminal nerve injury

## Abstract

Trigeminal nerve injuries are among the most challenging complications encountered in oral and maxillofacial surgery, often resulting in significant sensory and functional deficits. The present narrative review explores the evolving paradigms in the diagnosis and management of these injuries, focusing on conventional microsurgical techniques as well as recent advancements in regenerative therapies. Common causes include surgical trauma, chemical insults, and implant-related complications. Diagnostic modalities such as qualitative and quantitative neurosensory testing, cone-beam computed tomography (CBCT), and magnetic resonance neurography are discussed. Management options range from early microsurgical intervention and nerve grafting to the use of nerve conduits, stem cells, growth factors, and electrical stimulation. Despite these advancements, anatomical complexity, timing of intervention, and individual biological variability pose significant limitations. Emerging technologies, including AI-based imaging, soft tissue-driven planning, and virtual surgical simulation, offer promising prospects for improving patient outcomes. The review emphasizes the need for timely, individualized, and multidisciplinary approaches to optimize functional recovery and quality of life.

## Introduction and background

Trigeminal nerve injury is a recognized yet often underappreciated complication associated with oral surgical procedures. These injuries fall under the broader category of subclinical iatrogenic insults, defined as damage to neural structures caused not by the disease itself but by interventions intended to treat or manage it [1]. Such injuries are particularly concerning due to their potential to disrupt sensory innervation in critical areas of the maxillofacial region.

The trigeminal nerve, or the fifth cranial nerve, is primarily responsible for sensory innervation of the face, oral mucosa, and associated structures. It comprises three major branches: the ophthalmic, maxillary, and mandibular divisions. Among these, the mandibular division, and, more specifically, its branches, such as the inferior alveolar nerve (IAN), the mental nerve, and the lingual nerve, are most frequently affected during oral and maxillofacial procedures [2,3]. These branches are highly vulnerable to trauma due to their anatomical course and proximity to common surgical sites. The common sites at risk of trigeminal nerve injury are listed in Table 1.

**Table 1:** Common areas at risk for trigeminal injuries BSSO: Bilateral sagittal split ostectomy.

Region	Risk Involved
Mandibular third molar region	Risk to lingual and inferior alveolar nerve
Mandibular premolar region	Risk to mental nerve
Ramus and body of mandible	During BSSO or trauma repair
Maxillary tuberosity	Posterior superior alveolar nerve insult during extractions
Palate	Greater palatine nerve

The etiology of trigeminal nerve injuries is multifactorial. Common causes include chemical insults during dental treatments, such as endodontic overfills or local anesthetic toxicity [4,5], surgical manipulation during osteotomy procedures [6-8], direct trauma during removal of impacted third molars or tumors, and mandibular fractures involving the body or parasymphysis, which can damage the IAN or mental nerve [9]. While disruption of surrounding soft tissues may result in transient edema, ischemia, or infection, direct insult to the nerve can lead to more severe consequences such as elongation, compression, maceration, or transection of the nerve fibers [10]. The different types of trigeminal nerve injuries are listed in Table 2.

**Table 2:** Types of trigeminal nerve injuries following oral surgery IAN: Inferior alveolar nerve; MSCs: Mesenchymal stem cells; ESS: Endoscopic sinus surgery.

Type of Injury	Definition	Clinical Features	Prognosis	Examples
Neuropraxia	Temporary conduction block without axonal disruption	Numbness or tingling; full recovery expected within weeks	Excellent; typically resolves in 4–6 weeks	Stretching or compression during third molar extraction
Axonotmesis	Disruption of axons with intact connective tissue sheaths	Paresthesia, dysesthesia; gradual recovery over months	Fair to good; recovery via axonal regeneration	Crush injury during implant placement or osteotomy
Neurotmesis	Complete severance of the nerve including connective tissue	Complete sensory loss; often with painful dysesthesia	Poor; requires surgical repair	Nerve transection during osteotomy or cyst enucleation
Compression neuropathy	Injury due to prolonged mechanical or chemical pressure	Dull, aching pain; sensory deficits; may worsen over time	Variable; may require decompression	Overfilling root canal sealer impinging on IAN
Chemical neurotoxicity	Damage from chemical agents in close proximity to nerve tissue	Burning pain, altered sensation, hyperalgesia	Variable; depends on exposure duration	Extrusion of irrigants or medicaments into IAN canal
Thermal injury	Nerve damage from heat generated by surgical instruments	Immediate sharp pain, followed by numbness or dysesthesia	Variable; may need microsurgical intervention	Use of high-speed drills without adequate irrigation

The clinical impact of trigeminal nerve injuries is considerable and extends far beyond localized numbness. Neural damage may result in transient sensory loss or progress to more debilitating conditions such as neuropathic pain or trigeminal neuralgia, both of which are associated with chronic and potentially life-altering morbidity [11,12]. Patients often present with a range of symptoms, including hypoesthesia, dysesthesia, or paresthesia in the orofacial region. These sensory disturbances are frequently accompanied by functional impairments such as pain during mastication, allodynia, or persistent discomfort during occlusion [13]. The cumulative effect of these deficits can significantly compromise a patient’s quality of life, impairing social interaction, nutritional intake, and psychological well-being.

Given the anatomical complexity of the trigeminal nerve and the diverse etiologies, presentations, and outcomes of its injuries, clinicians are often faced with diagnostic uncertainty and therapeutic dilemmas. Although several traditional surgical approaches have been developed over the years, their outcomes remain inconsistent, and they are often limited by delayed referrals, technical challenges, and variable nerve regeneration capacities. Furthermore, the rapid evolution of regenerative and adjunctive therapies demands continuous updating of clinical knowledge and practice. Despite the growing body of literature, there remains a lack of consolidated guidance for clinicians on integrating emerging strategies with conventional approaches. Therefore, the present review aims to provide a comprehensive synthesis of the current paradigms in the diagnosis and management of trigeminal nerve injuries following oral surgery. It seeks to highlight established methods, examine recent innovations, and explore future directions, thereby equipping clinicians and researchers with an updated framework for optimizing outcomes in affected patients.

## Review

Methodology

This narrative review was conducted with the goal of synthesizing current evidence on the diagnosis and management of trigeminal nerve injuries associated with oral and maxillofacial surgical procedures. To ensure scientific rigor of the review, the methodology was guided by the principles of the SANRA (Scale for the Assessment of Narrative Review Articles), which evaluates six domains, including justification of the review, literature search strategy, referencing, scientific reasoning, appropriate presentation of data, and objectivity of interpretation.

A comprehensive electronic literature search was carried out using the databases PubMed, Scopus, Web of Science, and Google Scholar from their inception until May 2025. The search strategy employed a combination of MeSH (Medical Subject Headings) terms and free-text keywords, including “trigeminal nerve injury,” “lingual nerve,” “inferior alveolar nerve,” “oral surgery,” “nerve regeneration,” “microsurgical repair,” “nerve conduits,” “stem cells,” and “iatrogenic nerve damage.” Boolean operators such as AND/OR were used to refine the search. Only peer-reviewed articles published in English were considered. Manual searches of the bibliographies of key articles were also performed to identify additional relevant literature.

The review was structured around a focused clinical question framed using the Population, Intervention, Comparison, and Outcome (PICO) format: Population-patients undergoing oral and maxillofacial surgical procedures; Intervention-diagnostic and therapeutic approaches for trigeminal nerve injuries; Comparison-conventional microsurgical techniques versus novel regenerative strategies; and Outcomes-nerve recovery, sensory restoration, and complications. The inclusion criteria encompassed clinical studies, animal research, consensus statements, and high-quality narrative or systematic reviews addressing the etiology, diagnosis, and treatment of injuries to the trigeminal nerve and its branches. Articles were included if they discussed at least one of the following: common sites of injury, neurosensory evaluation techniques, imaging modalities, microsurgical repair methods, nerve grafting, or emerging regenerative techniques such as biomaterials, stem cells, and neurotrophic factors.

**Figure 1:**
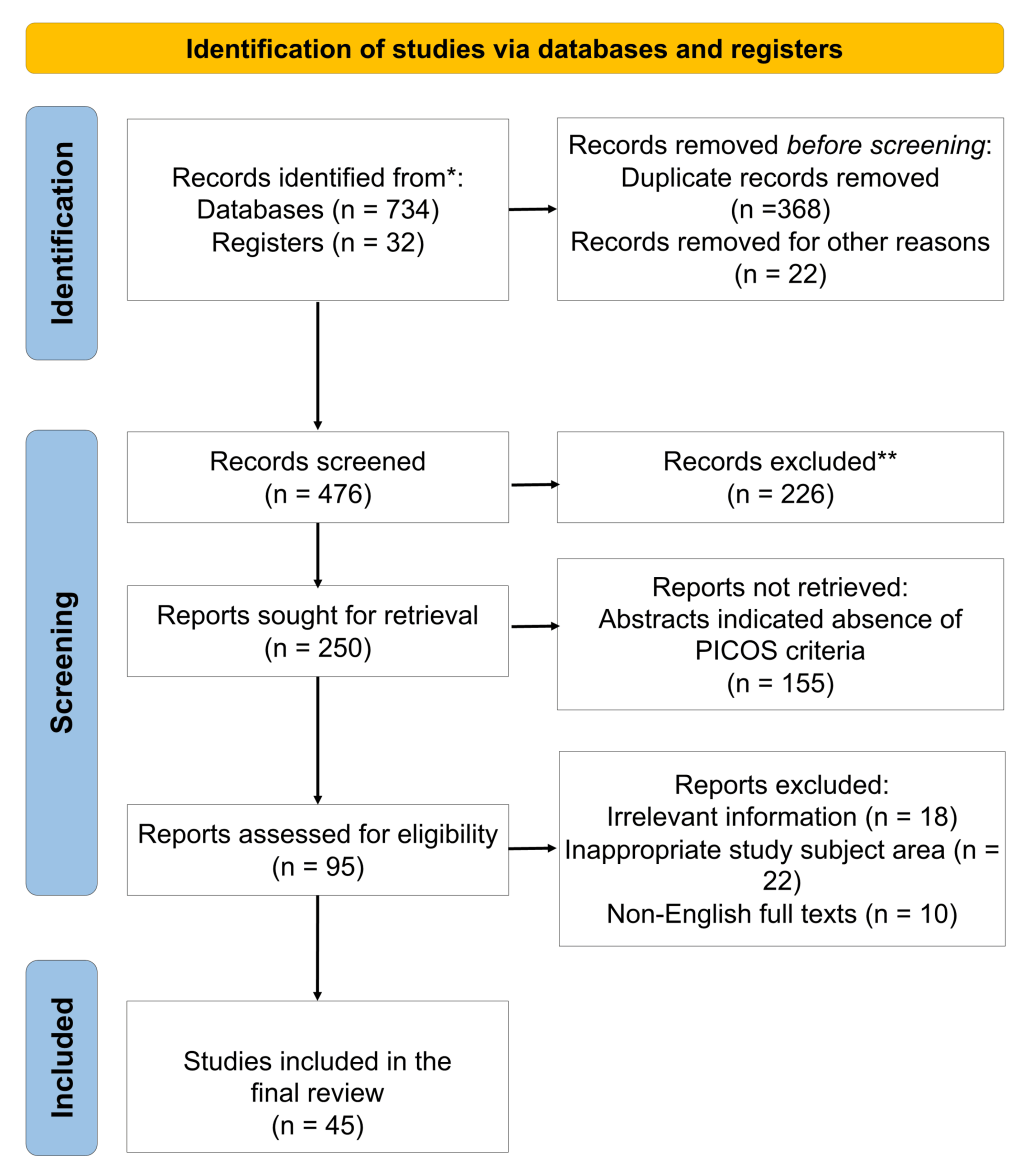
Flow diagram indicating the selection process of the article in the present narrative review PICOS: Population, Intervention, Comparison, Outcomes, and Study. *Databases including PubMed, Scopus, Web of Science, and Google Scholar. **Articles not satisfying PICOS criteria.

Studies were excluded if they focused on regions apart from the oral and maxillofacial region, were not available in English, lacked relevance to clinical or biological aspects of nerve injury, or were purely opinion-based without supporting evidence. Key studies were selected based on their relevance, methodological quality, and contribution to understanding evolving paradigms in diagnosis and treatment. Reference mining was also undertaken to identify additional landmark articles. All eligible studies were critically reviewed and grouped thematically under major categories, including anatomical susceptibility, diagnostic protocols, management strategies, limitations, and future innovations. Data were summarized thematically across categories such as etiology, clinical presentation, diagnosis, management strategies, microsurgical techniques, nerve repair materials, and emerging technologies. No meta-analysis or quantitative synthesis was performed, as the aim of this review was to provide a qualitative overview and expert interpretation of current trends. The study selection process is depicted in Figure 1.

Diagnosis

The accurate diagnosis of trigeminal nerve injuries is fundamental to determining prognosis and guiding timely intervention [14]. According to a consensus study conducted by Van der Cruyssen et al. [15], bedside clinical examination using qualitative neurosensory testing (QST) is considered the gold standard for initial assessment. This includes tests for light touch, pin-prick sensation, two-point discrimination, and directional sense. These evaluations provide a rapid yet informative overview of the sensory impairment. Following this, lesions are typically graded using the Medical Research Council Scale (MRCS), which allows clinicians to classify the severity and monitor changes over time. In cases where qualitative results are ambiguous or inconsistent, the use of QST, involving objective measurements of thermal and mechanical thresholds, is recommended to refine the diagnosis and document the progression of sensory recovery.

Imaging techniques also play a supplementary role in the diagnostic process, especially for preoperative risk assessment and localization of nerve injury. Although cone-beam computed tomography (CBCT) is widely used in dental practice, its diagnostic yield for soft tissue nerve injuries is limited. Nonetheless, it remains essential to evaluate the proximity of the inferior alveolar canal to third molar roots or implants, which is particularly useful in presurgical planning to mitigate nerve injury risk [16]. In high-risk cases, procedures such as coronectomy, removal of the crown while retaining the roots, are recommended to minimize the risk of IAN damage during third molar extractions. Similarly, panoramic radiographs (panorex) and CBCT are routinely employed to assess the spatial relationship between the implant site and the mandibular canal during implant placement. Accurate radiographic evaluation helps in identifying potential hazards such as canal perforation or cortical plate thinning that may predispose to nerve trauma.

For soft tissue visualization and postoperative assessment, magnetic resonance neurography (MRN) has emerged as a valuable modality. MRN provides detailed imaging of peripheral nerve architecture, enabling the identification of neuromas, scar tissue, or disruption of fascicular patterns [15]. However, the utility of magnetic resonance imaging (MRI) in general for trigeminal nerve injuries remains inconclusive and is not uniformly adopted across clinical practices [16].

A common scenario of trigeminal nerve damage occurs during implant placement, where mechanical trauma from drills or implants, thermal injury, or tissue retraction can compromise the IAN. Prevention in such cases hinges on thorough preoperative imaging and planning, as well as intraoperative vigilance. Postoperative imaging may also be warranted when patients report persistent altered sensations following surgery in order to detect possible nerve impingement or compression by the prosthetic components [14].

Management of nerve injuries

Timely intervention plays a pivotal role in the successful management of trigeminal nerve injuries. Among all treatment variables, the timing of surgical repair remains the most critical determinant of favorable outcomes. When the IAN is compromised during root canal treatment, microsurgical repair should ideally be performed within 48 hours to prevent permanent deficits [14]. Similarly, if nerve injury occurs during implant placement, the implicated implant should be removed as promptly as possible. In cases where immediate surgery is not warranted, a conservative approach involving monthly neurosensory testing is advocated. This serial testing enables clinicians to monitor the progression of spontaneous recovery and make informed decisions regarding the necessity of surgical exploration or repair [14]. Persistent sensory disturbances without signs of improvement over time are indicative of more serious nerve disruption and may require minor surgical intervention [16]

Clinical practice recommendations following trigeminal nerve injury during oral surgery

**Figure 2:**
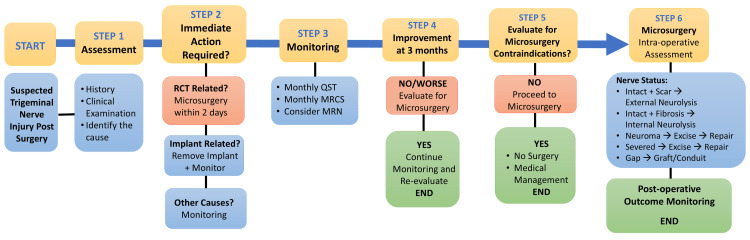
Treatment planning for a case of trigeminal neuralgia RCT: Root canal treatment. Image credits: Saanvi Tank and Sanpreet Singh Sachdev.

A thorough clinical history and structured neurosensory examination form the cornerstone of treatment planning. If no sensory recovery is evident by three months post-injury, particularly in response to directional sensitivity, soft touch, pin-prick, or two-point discrimination, microsurgical intervention is considered appropriate [14]. Numerous factors affect this decision-making process. The interplay between the various factors affecting the treatment planning for a case of trigeminal neuralgia occurring post-surgery is depicted in Figure 2.

In the unfortunate event of a trigeminal nerve injury during oral and maxillofacial surgical procedures, early identification and timely intervention are critical to optimizing patient outcomes and minimizing long-term sequelae [9,10]. Clinicians should begin with a thorough documentation of the injury, including the precise location, nature of the procedure, instrument used, and intraoperative observations. Immediate postoperative assessment should involve structured neurosensory testing to evaluate the presence of paresthesia, anesthesia, or dysesthesia [15]. If altered sensation persists beyond four to six weeks, patients should be referred to a specialist for advanced neurosensory evaluation, ideally including QST and imaging such as MRN to localize the lesion and assess nerve continuity [15,16]. Conservative management with close observation is appropriate for minor neuropraxic injuries showing early improvement. However, if no sensory recovery is noted by 8-12 weeks or symptoms worsen, surgical exploration should be considered [17,18]. Patient counseling is imperative throughout the process to manage expectations and ensure informed decision-making [14]. Multidisciplinary collaboration with neurologists, pain specialists, and oral surgeons is strongly encouraged for complex cases [11,12]. Furthermore, preventive strategies including proper anatomical knowledge, surgical planning, and avoidance of excessive force or deep penetration, particularly in high-risk zones such as the third molar, mandibular premolar, and maxillary tuberosity regions, remain essential in mitigating nerve injury risk [2,3,5].

Treatment options

A wide range of treatment modalities has been explored for the management of trigeminal nerve injuries, varying from well-established microsurgical interventions to novel regenerative approaches. Table 3 summarizes the principal treatment options along with their current status in terms of clinical applicability.

**Table 3:** Summary of treatment options for trigeminal nerve injuries and their clinical application status MSCs: Mesenchymal stem cells; iPSCs: Induced pluripotent stem cells; NGF: Nerve growth factor; BDNF: Brain-derived neurotrophic factor; VEGF: Vascular endothelial growth factor; PGA: Polyglycolic acid; LLLT: Low-level laser therapy.

Treatment Modality	Description	Current Clinical Application Status
Microsurgical repair (neurorrhaphy, neurolysis, and neuroma excision)	Includes external/internal neurolysis, coaptation of severed nerve ends, and neuroma resection	Established clinical practice
Autologous nerve grafting	Donor nerve (e.g., sural or auricular) used to bridge nerve gaps greater than 5 mm	Gold standard in clinical use
Nerve transfers	Functional donor nerve redirected to reinnervate the affected region	Used clinically in selected severe cases
Free vascularized nerve grafts	Graft with vascular pedicle to maintain blood supply and reduce fibrosis	Used clinically with limited availability
Synthetic/biological nerve conduits (e.g., silicone, collagen, and PGA)	Tubular scaffolds used to bridge short nerve gaps (≤10 mm)	Approved for clinical use in short-gap repair
Vein-muscle conduits	Autogenous vein filled with skeletal muscle to support regeneration	Preclinical and limited human trials
Biodegradable polymer tubes	Scaffolds with embedded filaments for guided nerve growth over longer gaps	Experimental (in vivo animal studies)
Collagen-based tubes	Collagen conduits supporting regeneration of short nerve defects	Approved and in clinical use
Schwann cell-based conduit fillers	Schwann cells embedded in hydrogels (e.g., alginate-fibronectin) to enhance axonal growth	Translational stage (preclinical)
Growth factor delivery systems	Use of neurotrophic factors (e.g., NGF, BDNF, and VEGF) to stimulate regeneration	Preclinical (in vivo animal studies)
Stem cell therapies	MSCs, iPSCs, or neural stem cells applied to enhance nerve regeneration	Investigational (translational/preclinical stage)
Electrical stimulation	Application of localized electrical pulses post-repair to accelerate regeneration	Investigational (animal studies)
Nanoparticle-enhanced scaffolds	Incorporation of nanosilver and laminin to promote myelination and conduction	Preclinical (experimental in vivo studies)
Bipolar radiofrequency therapy	Minimally invasive technique for modulating nerve repair	Early-stage investigation
LLLT	Adjunctive therapy used with grafts to improve fiber maturation and healing	Limited clinical studies; further validation needed

Trigeminal Nerve Microsurgery

Microsurgical intervention is generally indicated when neurosensory dysfunction persists for at least three months, suggesting that spontaneous recovery is unlikely. The presence of dysesthesia or worsening hypoesthesia further supports the need for surgery, especially when symptoms interfere with daily functioning or quality of life [17]. However, microsurgical repair is contraindicated in patients with central neuropathic pain, improving sensory function, well-tolerated hypoesthesia, or a prolonged lapse after the initial trauma. Medical comorbidities may also preclude surgical eligibility [18].

When the nerve remains anatomically intact but is tethered by fibrotic adhesions, external neurolysis is recommended. This involves the meticulous removal of surrounding scar tissue to release the nerve and facilitate regeneration [14,18]. In rare cases of intraneural fibrosis, internal neurolysis may be performed, which entails incising the epineurium to decompress the fascicles [18]. In cases of complete nerve transection, neurorrhaphy is the preferred technique. The injured segment is excised, and the proximal and distal nerve stumps are re-approximated using microsutures, often with a biological nerve protector to enhance healing [14].

In instances of neuroma formation, the neuroma is excised, and both nerve ends are inspected for viable fascicular architecture. If healthy tissue is identified, the ends are brought together with epineurial sutures. If tension-free coaptation is not achievable, blunt dissection is used to gain sufficient mobility of the nerve stumps [18].

Nerve Repair Techniques

In cases where the nerve continuity is disrupted and a tension-free primary repair is not possible, various nerve repair techniques are employed to restore function. These include nerve grafting, nerve transfers, vascularized nerve grafts, and synthetic or biological conduits.

Nerve grafting: When the nerve gap exceeds 5 mm and cannot be closed without undue tension, autologous nerve grafting remains the gold standard for repair. In this procedure, a donor nerve, commonly the sural or greater auricular nerve, is harvested and used to bridge the defect. While effective, autografting carries drawbacks such as limited donor availability, anatomical mismatch, neuroma formation, and donor site morbidity [19].

Nerve transfers: Nerve transfers are considered in severe proximal nerve injuries where conventional grafting is unlikely to yield functional outcomes. This method involves redirecting a less critical donor nerve to reinnervate the denervated target. Although historically challenging due to the slow pace of regeneration and early motor end plate atrophy, nerve transfers offer a viable alternative when primary or graft-based repair is not possible [20].

Free vascularized nerve grafts: One limitation of conventional nerve grafts is delayed revascularization, which can lead to central necrosis and failure of axonal regeneration. Free vascularized nerve grafts address this issue by preserving the vascular pedicle and anastomosing it to recipient vessels, thus maintaining continuous blood flow. This approach reduces fibroblast infiltration, supports Schwann cell (SC) survival, and minimizes endoneural scarring, thereby enhancing the rate and quality of regeneration [21].

Nerve conduits: To avoid harvesting native nerve tissue, synthetic and biological conduits are being increasingly utilized. Materials such as silicone, collagen, and polyglactin (PGA) mesh have been tested in clinical and experimental settings. These conduits create a protected environment for axonal regrowth across the nerve gap. However, conduit efficacy is typically limited by the maximum gap they can bridge while maintaining functional recovery, often cited as ≤10 mm in clinical settings [22].

Vein-muscle conduits: Experimental models, particularly in the rat sciatic nerve, have demonstrated that muscle-vein combination grafts can effectively promote nerve regeneration over distances up to 3 cm. Within 14 days post-surgery, substantial nerve fiber ingrowth has been observed, and by six months, the regenerated nerves exhibited significantly higher myelinated fiber density compared to controls. However, the average fiber size remained smaller, suggesting partial regeneration [23].

Biodegradable polymer tubes: Artificial grafts incorporating microfilament scaffolds, such as polyamide filaments within silicone tubes, have been tested in bridging longer nerve gaps (up to 15 mm). These tubes support axonal guidance while providing a temporary scaffold that eventually biodegrades. Early positive outcomes in animal models showed complete axonal growth within the conduit and functional recovery supported by neurofilament staining and sensory testing [24]. The resorbable nature of these scaffolds eliminates the need for secondary surgical removal [25].

Collagen tubes: Collagen-based conduits have shown comparable results to autografts in primate studies. These tubes support physiological nerve repair and have demonstrated success in rodents, rabbits, and nonhuman primates. Studies report that nerve gaps up to 5 mm can be effectively bridged with collagen nerve guides, reinforcing their clinical potential [26,27].

Nerve Regeneration Techniques

In recent years, significant advancements have been made in the field of peripheral nerve regeneration, offering adjunctive solutions to improve outcomes in trigeminal nerve repair. These techniques are primarily based on bioengineering principles and cellular therapies that aim to stimulate or accelerate axonal regrowth through biochemical, cellular, or physical modulation.

Conduit lumen fillers: One of the most promising strategies to enhance conduit performance is the use of SCs, which are essential for peripheral nerve repair due to their ability to promote axonal elongation and remyelination. Mosahebi et al. demonstrated that embedding SCs within an alginate hydrogel matrix, either with or without fibronectin, enhanced cell viability and regenerative capacity [28]. The addition of fibronectin further improved SC proliferation and axonal support. This synergistic combination not only preserved cell viability but also significantly increased the neurotrophic effect within the bioengineered conduit [28].

Growth factors: A key limitation in nerve conduit technology is the lack of sustained neurotrophic stimulation. To address this, various growth factors have been incorporated into scaffolds to promote regeneration. These include survival motor neuron-derived factor (SMDF), mechano-growth factor (MGF-1), vascular endothelial growth factor (VEGF), basic fibroblast growth factor (FGF-2), nerve growth factor (NGF), brain-derived neurotrophic factor (BDNF), glial cell-derived neurotrophic factor (GDNF), ciliary neurotrophic factor (CNTF), and neurotrophins NT-3, NT-4, and NT-5. These biomolecules enhance axonal sprouting, support SC activity, and contribute to the reestablishment of functional neural circuits [29,30].

Stem cell-based therapies: The limited availability of activated glial cells post-injury has led to the exploration of stem cell therapies. Neural stem cells, mesenchymal stem cells (MSCs), and induced pluripotent stem cells (iPSCs) serve as biologically active sources that supplement SC functions. MSCs, in particular, aid in nerve repair through the secretion of neurotrophic factors and their ability to transdifferentiate into glial-like cells that integrate into the regenerating axonal environment. These properties make stem cells a potent adjunct in the enhancement of both myelination and functional recovery following nerve trauma [29,31].

Electrical stimulation: Electrostimulation has emerged as a physical modality to enhance the speed and efficiency of nerve regeneration. Brushart et al. reported that a single one-hour session of 20-Hz electrical stimulation in a rat femoral nerve model significantly accelerated the process of axonal elongation. This approach helps overcome the natural "staggered regeneration" pattern observed after nerve transection, engaging motor neurons more synchronously and expediting functional reinnervation [31].

Nanoparticles: The integration of nanotechnology in nerve repair has shown encouraging outcomes. Biodegradable scaffolds composed of collagen type I and gelatin, impregnated with laminin and nanosilver particles, have been used to promote axonal regrowth. The nanosilver-collagen complex was found to enhance myelin sheath thickness, improve conduction velocity, and amplify nerve impulse amplitude, thereby reinforcing the overall regenerative milieu [28].

Bipolar radiofrequency: Bipolar radiofrequency (bRF) therapy, though still in its early investigational stages, has shown potential in inducing early nerve fiber degeneration followed by regeneration. It has recently been combined with micro-tenotomy and extracorporeal shock wave therapy (ESWT) as part of minimally invasive nerve modulation protocols. Early results indicate potential benefits in sensory nerve remodeling, although long-term efficacy remains to be confirmed [32].

Low-power laser application: Laser-assisted nerve regeneration represents another innovative frontier. Studies indicate that when a low-level laser is applied in conjunction with fat-enriched vein grafts, there is significant improvement in myelin sheath thickness, nerve fiber area, and axonal diameter compared to vein grafts alone. This combination appears to enhance both structural and functional nerve repair and may serve as a viable adjunct in clinical settings [25].

**Figure 3:**
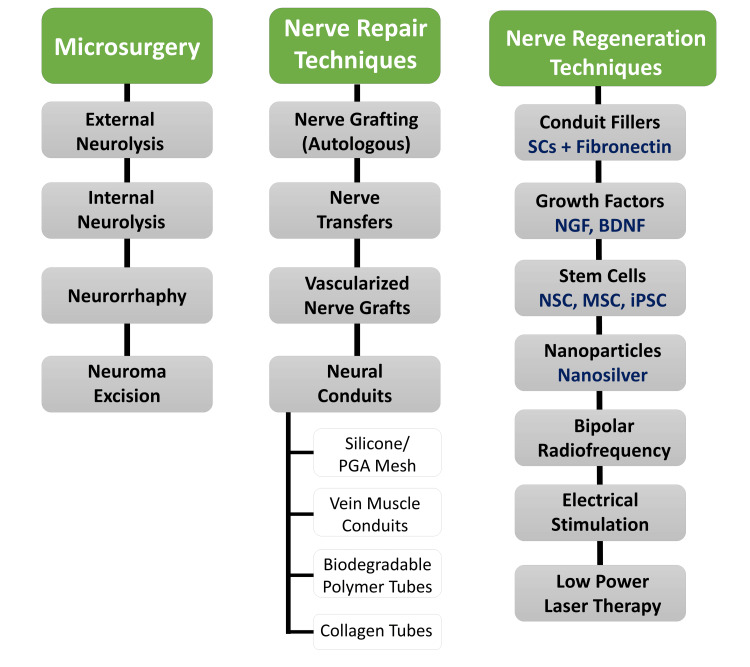
Treatment strategies available for trigeminal neuralgia PGA: Polyglycolic acid; SCs: Stem cells; NGF: Nerve growth factor; BDNF: Brain-derived neurotrophic factor; NSC: Neural stem cell; MSC: Mesenchymal stem cell; iPSC: Induced pluripotent stem cells. Image credits: Saanvi Tank and Sanpreet Singh Sachdev.

Overall, the various treatment strategies available for trigeminal neuralgia are collectively displayed in Figure 3.

Limitations

Despite significant advancements in both surgical and regenerative techniques, the management of trigeminal nerve injuries remains fraught with challenges. These limitations arise from a combination of anatomical, biological, and clinical complexities that influence treatment outcomes. One of the most prominent hurdles is the anatomical complexity of the trigeminal nerve. Its intricate, tripartite branching pattern and its close association with critical facial structures render it a highly delicate structure to access and repair surgically. The nerve’s coiled and deep-seated course, particularly in the mandible and pterygomandibular region, increases the risk of inadvertent damage during surgical intervention or incomplete restoration following trauma [33]. This complexity also complicates visualization, manipulation, and realignment of injured nerve segments during microsurgical procedures.

The timing of intervention is another critical determinant of success. Delayed diagnosis or referral can result in progressive nerve degeneration, fibrosis, and the formation of neuromas, which greatly diminish the chances of functional recovery. Unfortunately, immediate intervention is not always feasible due to comorbid medical conditions, postoperative inflammation, or delayed recognition of the injury. These constraints may result in missed windows of optimal surgical timing [34]. The severity and extent of the nerve injury also significantly affect prognosis. Clean, partial injuries are more amenable to repair compared to complete transections or crush injuries, which often involve extensive axonal and connective tissue damage. In cases of major nerve disruption, the chances of achieving full sensory or functional recovery remain limited, even with advanced techniques [35].

Although microsurgical methods have evolved considerably, they are still constrained by technical limitations. Precise coaptation of individual fascicles requires highly skilled surgical expertise and microsurgical infrastructure, which may not be universally available. Moreover, even well-executed repairs can be compromised by postoperative scar formation that impedes axonal regeneration or results in entrapment of regenerating fibers [36]. Biological variability further complicates outcomes. Factors such as the patient’s age, immune status, systemic health, and intrinsic healing capacity play a substantial role in determining the regenerative potential of the injured nerve. In many cases, even with optimal intervention, the nerve may not fully regain its original sensory or functional profile [37].

Another important consideration is the unpredictability of functional outcomes and patient-specific responses. Some patients may achieve near-complete sensory restoration, while others continue to experience persistent dysesthesia, numbness, or neuropathic pain despite successful anatomical repair. This variability underscores the individualized nature of nerve healing and complicates standardized treatment planning [38]. Beyond the physiological domain, the psychological burden of nerve injury is often under-recognized. Chronic paresthesia, altered facial sensation, or neuropathic pain can profoundly impact a patient’s emotional well-being, social functioning, and quality of life. These psychological dimensions often require multidisciplinary management, extending beyond the scope of surgical repair [39]. Finally, the risk of re-injury or postoperative complications remains a persistent concern. Infections, hematoma formation, or local swelling can compromise the integrity of nerve repairs. Moreover, future surgeries in the same anatomical region may inadvertently traumatize the previously repaired nerve, nullifying the progress made [40-44].

Current clinical evidence

A consolidated summary of key studies supporting diagnostic approaches and therapeutic interventions in trigeminal nerve injury management from the individual articles identified during the literature review is presented in Table 4.

**Table 4:** Summary of key referenced studies on the diagnosis and management of trigeminal nerve injuries DO: Distraction osteogenesis; BSSO: Bilateral sagittal split osteotomy; CBCT: Cone-beam computed tomography; IAN: Inferior alveolar nerve; PGA: Polyglycolic acid; QST: Qualitative sensory testing; MRCS: Medical Research Council Scale; MR: Magnetic resonance; VR: Virtual reality; 3D: Three-dimensional.

Author(s)	Year	Study Type	Key Findings/Methodology
Jerjes et al. [2]	2010	Clinical study/review	Identified risk factors for IAN and lingual nerve injuries during third molar removal.
Pogrel and Kaban [3]	1993	Review	Analyzed causes and nature of IAN damage during root canal therapy.
Wijbenga et al. [6]	2009	Clinical study	Compared neurosensory outcomes in DO vs. BSSO; highlighted differential recovery patterns.
Bagheri et al. [9]	2009	Clinical study/review	Reviewed microsurgical repair outcomes in maxillofacial trauma-related trigeminal nerve injuries.
Van der Cruyssen et al. [15]	2023	Consensus statement	Recommended QST and MRCS for diagnosis; QST and MR neurography for uncertain cases.
Schiavone and Ziccardi V [16]	2021	Literature review	Provided a comprehensive synthesis of trigeminal nerve injuries in oral surgery.
Ziccardi and Steinberg MJ [17]	2007	Literature review	Identified three-month non-recovery period as optimal threshold for considering microsurgery.
Ziccardi [18]	2011	Technique review	Described microsurgical techniques including neurolysis, neurorrhaphy, and neuroma excision.
Nectow et al. [19]	2012	Review	Discussed biomaterials (collagen, silicone, and PGA) and future strategies for nerve conduits.
Nath and Mackinnon [20]	2000	Review	Discussed indications and limitations of nerve transfers for proximal nerve injuries.
Strauch [22]	2000	Review	Evaluated use and limitations of nerve conduits in clinical and experimental repair.
Battiston et al. [23]	2000	Animal study	Vein-muscle grafts enhanced nerve fiber density over six months in rat sciatic models.
Mosahebi et al. [28]	2003	Experimental study	Fibronectin-enhanced alginate matrix improved Schwann cell viability and regeneration.
Brushart et al. [31]	2002	Animal study	20-Hz electrical stimulation post-suture enhanced motoneuron regeneration in rats.
Sarwar and Jabin [41]	2023	Review	AI-enhanced CBCT improves nerve visualization and risk assessment.
Fang et al. [33]	2023	Methodology study	Proposed soft tissue-driven planning to improve precision in craniofacial nerve surgery.
Nguyen et al. [42]	2023	Methodology study	Developed 3D auto-segmentation tools for surgical wound planning in nerve repair.
Vizziello et al. [43]	2023	Feasibility study	Demonstrated intra-body device communication for nerve monitoring and control.
Sadeghnejad et al. [44]	2019	Validation study	Validated VR haptic simulation for training in trigeminal nerve microsurgery.

Future prospects

The future of trigeminal nerve repair, especially in the context of oral surgical trauma, appears promising with several transformative innovations on the horizon. One such advancement is the emergence of soft tissue-driven surgical planning, which emphasizes the dynamic interplay between facial soft tissues and underlying skeletal structures. This approach is reshaping preoperative strategies, allowing for more precise identification and preservation of nerve pathways during surgical intervention [33].

Another exciting frontier is the integration of artificial intelligence (AI) in dental imaging. AI-enhanced diagnostic platforms are significantly improving the interpretation of CBCT scans, enabling clinicians to detect nerve proximity, deviations, or pathology with greater accuracy. This technology has proven invaluable in treatment planning and predicting potential complications related to trigeminal nerve involvement [41]. Further innovations are being seen in wound regeneration and management. Computerized systems now allow for the automatic delineation and three-dimensional analysis of facial wounds. These systems assist surgeons in planning nerve repairs by providing real-time visualization of soft tissue dynamics and surgical impact zones, thereby optimizing both function and aesthetics [42].

In parallel, research is being directed toward intra-body communication systems, which aim to create real-time interconnectivity between medical devices implanted within the human body. These systems may enable dynamic monitoring and responsive adjustments in nerve healing or regeneration, thereby personalizing postoperative management of nerve injuries [43]. Additionally, virtual reality (VR) simulation is revolutionizing surgical training. VR platforms that simulate microsurgical procedures with realistic haptic feedback are allowing clinicians to practice fine motor techniques in a controlled environment. This technology holds particular promise for mastering delicate interventions such as trigeminal nerve repair, where surgical finesse directly influences outcomes [44].

## Conclusions

Despite remarkable advances in our understanding and management of trigeminal nerve injuries, these conditions remain among the most intricate challenges in oral and maxillofacial surgery. Successful treatment requires an interdisciplinary approach that combines early diagnosis, precise microsurgical techniques, and regenerative adjuncts. Although microsurgical repair and evolving biomaterials have shown encouraging outcomes, limitations related to timing, anatomy, and individual variability persist. Novel technologies, including stem cell therapies, nerve conduits, electrical stimulation, and AI-driven diagnostics, are paving the way toward more personalized and minimally invasive interventions. This review underscores the importance of continued research and technological integration in optimizing patient-specific care. Ultimately, enhancing the predictability and success of trigeminal nerve repair is essential not only for restoring function but also for improving patients’ overall quality of life.
